# Subjective mental well-being among higher education students in Finland during the first wave of COVID-19

**DOI:** 10.1177/14034948221075433

**Published:** 2022-02-22

**Authors:** Kiira K. Sarasjärvi, Pia H. Vuolanto, Pia C.M. Solin, Kaija L. Appelqvist-Schmidlechner, Nina M. Tamminen, Marko Elovainio, Sebastian Therman

**Affiliations:** 1 Faculty of Medicine, Doctoral Programme in Population Health, University of Helsinki, Helsinki, Finland; 2 Faculty of Social Sciences, Research Centre for Knowledge, Science, Technology and Innovation Studies (TaSTI), University of Tampere, Tampere, Finland; 3 Finnish Institute for Health and Welfare, Mental Health Team, Helsinki, Finland; 4 Faculty of Medicine, Department of Psychology and Logopedics, University of Helsinki, Helsinki, Finland

**Keywords:** Mental well-being, SWEMWBS, COVID-19, first wave, higher education, Finland, cross-sectional

## Abstract

**Aims:**

Increased mental health problems during the COVID-19 pandemic have become a major concern among young adults. Our aim was to understand which COVID-19-related questions predicted mental well-being during the outbreak.

**Methods:**

Two cross-sectional datasets were used. The primary dataset was collected in May 2020 (*n* = 1001), during the initial COVID-19 outbreak, and the secondary in April 2019 (*n* = 10866), before the pandemic. Mental well-being was assessed with the Short Warwick–Edinburgh Mental Well-Being Scale. Relationships between mental well-being and COVID-19-related questions were investigated with lasso regression. As an exploratory analysis, two-way ANOVAs were used to compare mental well-being before and during the outbreak.

**Results:**

Higher levels of mental well-being were associated with lower levels of academic stress and COVID-19-related worry, along with a higher satisfaction with the procedures and information provided by the higher education institutions and the government. COVID-19-related symptoms and infections did not have an impact on students’ mental well-being during the outbreak. Small to moderate effect sizes across the time points were detected, indicating an overall decrease in mental well-being across age and gender during the outbreak.

**Conclusions:**

**COVID-19 had an impact on higher education students’ mental well-being. Higher education institutes may play a crucial role in protecting their students’ well-being during uncertain times.**

## Background

On the 11^th^ of March in 2020, the World Health Organization announced the Coronavirus disease (COVID-19) as a global pandemic [1]. In Finland, The Emergency Power Act was in place for 3 months (16 March to 15 June 2020) [2]. During this time, gatherings greater than 10 were banned, public places shut down, restaurants were open only for take-aways, and people began working and studying remotely [3]. In May 2020, childcare and primary schools reopened to their pupils, while higher education institutes remained shut [3].

A number of cross-sectional studies have reported relatively high rates of mental health-related symptoms (e.g., stress, depression, anxiety) during the early stage of the pandemic among general [4–6] and student [7,8] populations. Being female, young (18–35 years), a student, having poor self-reported health, and increased exposure to COVID-19-related news, experiencing COVID-19-related symptoms, or having an acquaintance infected by the virus were all factors associated with poor mental health [4–8]. Furthermore, academic stress, institutional dissatisfaction and fear of being infected by COVID-19 were associated with depressive symptom severity among students in Belgium [9]. In contrast, a positive attitude in general and towards protective measures (e.g. hand-washing), accurate information regarding the virus, and safety measures were associated with a lower psychological impact of the pandemic [5].

So far, only a few studies have longitudinally investigated differences in mental health before and during the pandemic [10–14]. In the UK, mental health-related symptoms and mental well-being have slowly increased and decreased, respectively, throughout the pandemic among general [11–13] and student [14] populations. In Germany, previous positive mental health and stress symptoms predicted a lower and higher burden during pandemic, respectively, which was mediated by perceived sense of control [10]. Surprisingly, previous depressive and anxiety symptoms did not have the same effect, emphasizing the importance of positive mental health during the pandemic [10].

Positive mental health is often defined as a state where an individual is feeling good and functioning well [15]. Mental well-being has been shown to protect college students from suicidal thoughts and learning difficulties [15]. Even before the pandemic, young adults and students were experiencing high levels of stress due to high study demands and a lack of social support that increased the risk of mental health-related symptoms [16]. Taking into consideration the previous stressors affiliated with student life, and the changes due to the COVID-19 pandemic, the current state of students’ mental health is of great concern.

## Aims

The primary aim was to investigate which COVID-19-related questions predicted mental well-being during the outbreak. We hypothesized that lower levels of mental well-being would be associated with COVID-19-related symptoms, infections, and higher academic stress as well as lower safety measure adherence and satisfaction with the education institute and government. As a secondary aim, we investigated the differences in mental well-being based on gender, age and education programme. Two cross-sectional datasets, before and during the pandemic, were used to gain a better understanding of the differences in mental well-being between the demographic groups. We expected to see lower levels of mental well-being during the pandemic than before. Based on the existing literature, we predicted that being female, younger age, and an undergraduate would be associated with lower levels of mental well-being at both time points.

## Methods

### Participants

The present study consisted of two cross-sectional datasets. The primary dataset was collected in May 2020 (11–27 May 2020) during the first outbreak in Finland as a part of a larger COVID-19 International Student Well-Being Study (C19 ISWS) [17]. The second dataset was collected in April 2019, 11 months prior to the pandemic. The participants gave their consent by completing the survey, after they were informed about the study data management. Participants who were not aged between 18 and 45, had incomplete surveys, or missing Short Warwick–Edinburgh Mental Well-being Scale (SWEMWBS) items were excluded from the analyses.

#### During COVID-19

The primary dataset is the result of a study design, study protocol, and questionnaire developed by a team at University of Antwerp, Belgium (Prof. Sarah Van de Velde, Dr. Veerle Buffel and Prof. Edwin Wouters) [17]. The multicountry research design was approved by the Ethics Committee for the Social Sciences and Humanities of the University of Antwerp and the Ethics Committee for the Social Sciences of Ghent University [17]. The study was conducted in 26 countries; only the Finnish sample is used in the present study because of the availability of two comparable datasets in this context. During data collection, the infection rates in Finland were decreasing (averaging 60 new cases a day) after the first peak in early April 2020 [18]. The anonymous online survey was shared with the students via email and social media by the universities, student associations, and other stake holders, using snowball sampling. The study had a total of 1374 respondents from 27 out of 37 Finnish higher education institutes, yielding a response rate of 0.5% among all students in the country. After exclusion of participants, the Finnish substudy yielded a total of 1001 participants.

#### Before COVID-19

The secondary dataset is a part of the Student Barometer administered by the Research Foundation for Studies and Education, Otus sr. According to the Finnish National Board on Research Integrity (TENK), the anonymous data collection did not require an ethical review statement for human sciences [19]. Personalized invitations and open links were sent via higher education institutes to their students who were enrolled for the academic year in 2019. The survey had a total of 11517 respondents from 36 out of 37 Finnish higher education institutes, yielding a response rate of 5.0%. The sample had a total of 10866 participants after exclusion criteria were met.

### Measures

#### Sociodemographics

Gender (male, female, other), age (18–25, 26–35, 36–45 year-olds) and education (bachelor’s-, master’s-, doctoral programme) were used to classify demographical background in both datasets.

#### Mental well-being

Mental well-being was measured in both datasets with the 7-item SWEMWBS [20], which is a shortened version of the 14-item WEMWBS [21]. The scale measures mental well-being within the last 2 weeks on a 5-point rating scale. The sum of the SWEMWBS responses are converted into a ‘metric score’ (range 7–35), with higher scores indicating higher mental well-being. The scale has been proven to be an adequate instrument for assessing mental well-being among general and student populations showing good internal consistency, high construct validity, and having a single-factor structure [20–22].

The primary dataset included several COVID-19-related questions:

**COVID-19-related symptoms and diagnosis:** Participants were asked whether they had (a) experienced any COVID-19-related symptoms in the past month or (b) whether they had had, thought they might have had, or if they knew someone with a COVID-19 infection.

**COVID-19-related worry:** Participants were asked five questions regarding how worried they were that (a) they, or (b) someone close to them would contract COVID-19, (c) they, or (d) someone else would get severely ill from it, or (e) that doctors and hospitals would not have sufficient medical supplies to handle the pandemic (0 = not worried, 10 = very worried).

**COVID-19 adherence:** Participants were asked how strictly they adhered to the protective measures during the outbreak (0 = not strictly, 10 = extremely strictly).

**Academic stress and satisfaction:** Participants were asked eight questions regarding changes in their studies due to the pandemic, and their opinion on how well their higher education institute had handled the COVID-19 situation. A five-point rating scale (0–4) was used, with higher scores indicating higher academic stress and satisfaction.

**Government satisfaction:** A five-point rating scale (0–4), higher scores indicating higher satisfaction, was used to assess how well the government had succeeded in providing (a) comprehensive and (b) topical information concerning COVID-19. The mean score on the two items was used as a measure of overall government satisfaction.

### Statistical analysis

As a preliminary analysis, three item factor analyses (IFAs) were conducted to ensure sufficient psychometric properties of the SWEMWBS, COVID-19-related worry, and academic stress and satisfaction scales (Supplementary Tables 10-12, 16-17, 20-21 for more details).

Percentages (%), means (*M*), and standard deviations (SD) were used to describe the distributions of all COVID-19-related questions, as well as the SWEMWBS scores. In further analyses, SWEMWBS metric scores were the dependent variable.

Lasso regression was chosen to investigate the association between COVID-19-related questions and mental well-being. Both the minimum lambda value and the stricter 1 standard error criterion were reported. COVID-19-related symptoms, infections, and worry, adherence to the restrictions, as well as academic stress and satisfaction, and government satisfaction, were used as predictors.

Exploratory two-way analyses of variance (ANOVAs) were conducted to investigate the main effects of the COVID-19 pandemic, demographic groups (gender, age, education), as well as their interactions. The two cross-sectional datasets were used as a grouping variable (time point) in all analyses. As a second predictor group we used gender, age and education programme, respectively. Bonferroni corrections were applied to the multiple comparisons of the post hoc tests. Cohen’s *d* and the common language effect size (CL) were used to indicate the magnitude of the change in mental well-being.

The effect sizes and lasso regression were calculated with the *orddom* v3.1 [23] and *glmnet* v4.1-1 [24] packages, respectively, in R 3.6.0 [25]. The IFAs were conducted with Mplus v8.3 [26]. All other analyses employed IBM SPSS Statistics 27 [27].

## Results

The characteristics and basic demographics of participants for both datasets are presented in Table I.

**Table I.** Participant demographics.

Measure	Before COVID-19 outbreak		During COVID-19 outbreak	
	*n*	%	*n*	%
Gender				
Male	2948	27.2	203	20.3
Female	7647	70.6	783	78.2
Other	240	2.2	15	1.5
Total	10835	100	1001	100
Age group				
18–25	5908	54.4	606	60.5
26–35	3884	35.7	297	29.7
36–45	1074	9.9	98	9.8
Total	10866	100	1001	100
Education programme				
Bachelor	7762	71.6	690	69.3
Master	2917	26.9	295	29.6
Doctoral	161	1.5	11	1.1
Total	10840	100	996	100

Note: Samples are cross-sectional.

Chi-square tests of independence between the samples for gender, age, and education yielded χ^2^(2, 11836) = 26.226, *p* < .001; χ^2^(2, 11836) = 15.959, *p* < .001; χ^2^(2, 11836) = 4.069, *p* = .131, respectively.

### Mental well-being and COVID-19 related questions

The majority (52.9%) had experienced possible COVID-19 symptoms, for example, coughing, sneezing, or a runny nose, within the month prior to the study, while 39.5% had not experienced any symptoms and 7.6% were unsure. The prevalence of COVID-19 infection was relatively small in our sample. Only 1.5% had contracted the virus, 12.9% knew someone who had, and 6.6% assumed they had contracted the virus. In total, almost a fifth (18.3%) either had been infected, thought they had been, or knew someone who had contracted COVID-19.

The majority of the students agreed that the COVID-19-related information provided by the government was comprehensive (74.9%) and current (71.1%). Also, adherence to the restrictions was relatively high (*M* = 7.74, SD = 1.72). However, the level of worry among students varied. Students were more worried about their acquaintances contracting the virus (*M* = 6.68, SD = 2.78) or getting sick (*M* = 7.14, SD = 2.69) than themselves (*M* = 4.27, SD = 2.78; *M* = 4.42, SD = 2.99, respectively).

Approximately 40% of the respondents were experiencing academic stress due to the COVID-19 pandemic, reporting increased workload (39.5%), uncertainty of what was expected from them (34.9%), and stress due to changes in study methods (41.0%). A third (33.5%) believed that the institute provided poorer education during the pandemic than before, and 27.5% were worried that they would be unable to finish the academic year due to COVID-19. However, 77.9% were satisfied with how their institute informed the students and implemented protective measures, though only 41% felt that they could speak to the staff regarding their concerns about COVID-19.

Our model explaining mental well-being during the pandemic had seven potential predictors related to COVID-19 (Table II). In the more liberal analysis, using the minimum lambda, the significant predictors of mental well-being during the COVID-19 outbreak were low academic stress (unstandardized coefficient −1.284), being less worried (−0.336) and being more satisfied with the higher education institute (0.389) and the government (0.206). Adherence to restrictions showed a very minimal (0.003) effect in the model. COVID-19-related symptoms or infections were not significant in our model. When using the stricter lambda value, only academic stress (−0.957) and satisfaction (0.022) remained significant predictors of mental well-being during the COVID-19 outbreak.

**Table II.** Unstandardized coefficients for lasso models predicting SWEMWBS metric scores during COVID-19 outbreak.

Predictor	Lambda minimum	Lambda 1 SE
(Intercept)	20.41	21.01
Academic stress F1 _(F)_	−1.284	−0.957
Academic satisfaction F2 _(F)_	0.389	0.022
Worry _(F)_	−0.336	.
Government _(Mean)_	0.206	.
Adherence _(O)_	0.003	.
COVID-19 symptoms _(N)_	.	.
COVID-19 infections _(N, D)_	.	.

Note: lambda minimum *R*^2^ = .175; lambda 1 SE (standard error) *R*^2^ = .129.

F: factor scores; O: ordinal scale; N: nominal scale; D: dichotomy.

### Mental well-being before and during the COVID-19 outbreak

As hypothesized, the overall subjective mental well-being was lower during the outbreak (*M* = 21.0, SD = 3.7) than before (*M =* 22.7, SD *=* 3.7). The analyses showed moderate effect sizes (*d* = 0.31–0.63, CL = 0.58–0.68) in all sociodemographic groups, except for PhD students (*d =* −0.07, CL = 0.52) (see Table III).

**Table III.** Means and standard deviations for SWEMWBS metric scores by socioeconomic demographics before and during the COVID-19 outbreak.

	Before COVID-19		During COVID-19		ANOVA			Effect sizes	
	*M* (SD)	95% CI	*M* (SD)	95% CI	Adj. mean (SE)	95% CI	*p-*value	*d*	CL
Total	22.7 (3.7)	[22.6, 22.8]	21.0 (3.7)	[20.8, 21.2]				−0.46	0.63
Gender					Levene’s test, *p* < .001		*p* < .001^a^		
Male	22.9 (4.0)	[22.7, 23.0]	21.4 (4.0)	[20.9, 22.0]	22.1 (.08)	[21.9, 22.2]		−0.36	0.60
Female	22.7 (3.5)	[22.6, 22.8]	20.9 (3.6)	[20.7, 21.2]	21.8 (.06)	[21.7, 22.0]		−0.49	0.63
Other	21.1 (3.7)	[20.6, 21.6]	18.8 (3.1)	[17.1, 20.5]	20.2 (.24)	[19.8, 20.7]		−0.63	0.69
Age group					Levene’s test, *p* = .32		*p* < .001^b^		
18 – 25	22.8 (3.6)	[22.7, 22.9]	20.9 (3.7)	[20.6, 21.2]	21.9 (.07)	[21.8, 22.0]		−0.51	0.64
26 – 35	22.3 (3.7)	[22.2, 22.4]	20.8 (3.5)	[20.4, 21.1]	21.5 (.08)	[21.3, 21.6]		−0.43	0.62
36 – 45	23.5 (3.8)	[23.3, 23.8]	22.3 (4.1)	[21.4, 23.1]	22.7 (.12)	[22.5, 23.0]		−0.33	0.59
Educ. programme					Levene’s test, *p* = .40		*p* = .42		
Bachelor’s	22.7 (3.7)	[22.6, 22.8]	21.1 (3.7)	[20.8, 21.4]	21.9 (.07)	[21.7, 22.0]		−0.44	0.62
Master’s	22.7 (3.7)	[22.5, 22.8]	20.8 (3.7)	[20.4, 21.3]	21.8 (.08)	[21.7, 22.0]		−0.50	0.64
Doctoral	22.9 (4.2)	[22.3, 23.5]	23.2 (3.7)	[20.7, 25.7]	22.2 (.29)	[21.6, 22.8]		0.08	0.48

Note: SWEMWBS metric scores. Effect sizes based on factor scores.

Values standardized by gender, age and education in Supplementary Table V.

a–b.Adjustment for multiple comparisons: Bonferroni.

a Gender: male > female (*p* = .006), male > other (*p* < .001), female > other (*p* < .001);

b Age: 18–25 > 26–35 (*p* < .001), 18–25 < 36–45 (*p* < .001), 26–35 < 36–45 (*p* < .001).

*M*: mean; SD: standard deviation; 95% CI: 95% confidence interval; Adj. mean (SE): adjusted mean (standard error); *p*-values based on the main effects; *d*: Cohen’s *d*; CL: common language effect size (probability X > Y).

The two-way ANOVAs did not show any significant interactions between the time points and sociodemographic variables. The main effect of the COVID-19 outbreak yielded a significant decrease in mental well-being after adjusting for gender, age, and education (Supplementary Table 5). The results revealed significant main effects for gender (*F*(2, 11832) = 31.00, *p* < .001) and age (*F*(2, 11863) = 54.69, *p* < .001), but not for education programme (*F*(2, 11832) = 0.87, *p* = .42), when mental well-being was adjusted for the time points.

Post hoc comparisons revealed significant differences in mental well-being across all gender (*p* < .01) and age (*p* < .001) comparisons. Mental well-being was the highest among males (*M =* 22.1, SD *=* 0.08) and the lowest in the non-binary group (*M =* 20.2, SD *=* 0.24). Moreover, the highest mental well-being was reported among 36–45 year-olds (*M =* 22.7, SD *=* 0.12), while the lowest mental well-being was found among 26–35 year-olds (*M =* 21.5, SD *=* .08).

## Discussion

The COVID-19 pandemic has raised great concerns about population mental health, especially among young adults. In the present study, our primary aim was to investigate which COVID-19-related topics were associated with higher education students’ mental well-being during the first outbreak in Finland. Furthermore, we compared these results to a similar student sample prior to the pandemic, in order to identify changes in mental well-being during the first period of the disease proliferation and restrictions. We discovered that greater mental well-being was strongly associated with how well students thought their education institutes had handled the outbreak. For example, lower levels of academic stress and higher levels of academic satisfaction were associated with higher mental well-being during the outbreak. Furthermore, both predictors remained statistically significant when using a stricter criterion, emphasizing the association between the higher education institute’s actions and students’ mental well-being during the outbreak. Furthermore, COVID-19-related worry was associated with lower mental well-being. Our results are similar to De Man and colleagues’ cross-sectional study [9], where they reported significant associations between depressive symptom severity, academic stress, institutional dissatisfaction, and COVID-19-related fear among Belgium higher education students.

Furthermore, trust in the government was associated with mental well-being: both being satisfied with the COVID-19-related information provided by the government, as well as higher adherence to the restrictions were associated with higher mental well-being. This result was expected, since a sense of control and being informed have been associated with mental health symptomology [10]. However, the effect of adherence to restrictions was relatively small. Previous studies have reported association between adherence and mental health in Europe and the US, also showing relatively small or mixed results [28,29]. Furthermore, the adherence among our student sample was high, and the small variance may have affected the results.

Contrary to previous findings from China [5,7,8], COVID-19 symptoms or infection did not predict mental well-being during the pandemic in our model. In general, the course of the pandemic has been less severe in Finland than in China. It is thus possible that students in Finland have not felt threatened by the virus, since the infection and overall mortality at the time were higher in older populations [18]. In fact, the prevalence of COVID-19 infection was very small in our sample, and the students were more worried about others being infected by the virus than themselves.

As in previous studies [11,12,14], the exploratory analyses revealed a significant decrease in overall mental well-being during the pandemic, along with higher mental well-being among male and older age groups for both time points. Like Savage and colleagues [14], we found no interaction effects on mental well-being between sociodemographic variables and time points. The lack of interaction may partly be explained by the homogeneity of the student sample. For example, significant interactions between gender, age, and time points were reported in the Welsh general population [11]. Additionally, we replicated the alarming finding of Smith and colleagues [4] regarding poorer mental well-being among non-binary students during the COVID-19 pandemic. Furthermore, the reduction in mental well-being was moderate across sociodemographic groups, except among PhD students. It is possible that PhD students’ lives were less disrupted, as their studies are much less dependent on in-person lectures and group assignments.

### Limitations

Our study had some limitations. Firstly, our study design prevented us from exploring longitudinal changes in mental well-being within individuals. However, by comparing our dataset to the similar cross-sectional sample from before the pandemic, we can report the levels of mental well-being before the pandemic that have often been left out in the literature.

Secondly, our sample is not fully representative of the Finnish student population: the majority identified themselves as female and studied in Eastern Finland. Similarly, the sample size for PhD students was relatively small. However, uneven group sizes are common in survey-based studies, and base proportions are obviously not equal either, for example, in 2019 in Finland 54% of students were female, whereas the proportion of doctoral students was approximately 10% [30]. Though some subgroups are rare, it is important to include these subgroups in future studies, not only to detect, but also understand the factors related to mental health within these minorities.

Thirdly, we used several unvalidated COVID-19-related questionnaires. Even though these scales provided more specific information about the pandemic, their psychometric validation is essential if we want to compare our findings among other student populations. Lastly, there might be some other student-specific characteristics outside the scope of our study (e.g., exam periods, part-time students, or students with children) that may have had an impact on our results.

## Conclusions

Our results revealed a decrease in mental well-being during the first COVID-19 outbreak, showing small to medium effect sizes. Overall, being satisfied with the higher education institute’s response seemed to play an important role in supporting the students’ mental well-being during the pandemic. It is therefore important that institutes provide information and support their students during these uncertain times.

## Supplemental Material

sj-docx-1-SJP-10.1177_14034948221075433 – Supplemental material for Subjective mental well-being among higher education students in Finland during the first wave of COVID-19 Click here for additional data file. Supplemental material, sj-docx-1-SJP-10.1177_14034948221075433 for Subjective mental well-being among higher education students in Finland during the first wave of COVID-19 by Kiira K. Sarasjärvi, Pia H. Vuolanto, Pia C.M. Solin, Kaija L. Appelqvist-Schmidlechner, Nina M. Tamminen, Marko Elovainio and Sebastian Therman in Scandinavian Journal of Public Health
