# Supplementary material for: Subjective mental well-being among higher education students in Finland during the first wave of COVID-19
Source: Scand J Public Health. 2022 Feb 22;50(6):765–71. doi: 10.1177/14034948221075433 (PMC9361411; doi:10.1177/14034948221075433)
Supplement: sj-docx-1-SJP-10.1177_14034948221075433 – Supplemental material for Subjective mental well-being among higher education students in Finland during the first wave of COVID-19 [file sj-docx-1-SJP-10.1177_14034948221075433.docx]

SUPPLEMENTARY MATERIALS

# Factor Analysis

In the SWEMWBS factor analysis, the two cross-sectional datasets were combined, yielding a total of 11 867 respondents. Due to the similarities between the metric, raw, and factor scores, metric scores were used in the present study as they had better psychometric properties than raw scores, but are easier to communicate and comprehend than factor scores.

The primary dataset (during COVID-19) was used to determine the factor scores for academic stress, satisfaction and COVID-19 related worry. Due to low frequencies of endorsing some response alternatives, the least frequently used response category on each scale was combined with the adjacent less extreme response category, and factor analyses used this recoded 1–10 scale.

The WLSMV estimator and Maximum A Posteriori (MAP) factor scores on the respective standardized latent factors (mean = 0 and variance = 1) were used in all factor analyses, with the items coded as categorical.

Based on the IFAs, one-factor models showed adequate fit for SWEMWBS (RMSEA = 0.139, CFI/TLI = 0**.**960 / 0.940) and COVID-19 related worry (RMSEA = 0.329, CFI/TLI = 0.958 / 0.915), respectively. Academic stress and satisfaction yielded a two-factor structure (RMSEA = 0.066, CFI/TLI = 0.986 / 0.970).

More detailed results (descriptiveness, intra-inter correlations, factor loadings, etc.) for SWEMWBS, Academic Stress and Satisfaction, COVID-19 related worry are presented in tables S6–13, S14–17, and 18–21, respectively.

# SWEMWBS Factor, Raw Scores of Lasso Regression and Two-Way ANOVAs (Tables 2 and 3)

## Supplementary Table 1. LASSO regression Predicting Mental Well-being with Academic Stress, Satisfaction, COVID-19 related worry, Satisfaction with the government, Adherence, and COVID-19 symptoms and infections

| Predictors | SWEMWBS Factor1 _(COM.)_ | |  | SWEMWBS Metric | |  | SWEMWBS Raw | |
| --- | --- | --- | --- | --- | --- | --- | --- | --- |
|  | *Lasso_min_* | *Lasso_1se_* |  | ***Lasso_min_*** | ***Lasso_1se_*** |  | *Lasso_min_* | *Lasso_1se_* |
| **Regression 1** |  |  | |  |  | |  |  |
| AC Stress F1 _(F)_ | -0.310 | -0.234 | | **-1.284** | **-0.957** | | -1.600 | -1.209 |
| AC Satisfaction F2 _(F)_ | 0.107 | 0.020 | | **0.389** | **0.022** | | 0.522 | 0.091 |
| Worry F1 _(F)_ | -0.085 | . | | **-0.336** | **.** | | -0.384 | . |
| Adherence scale _(O)_ | . | . | | **0.003** | **.** | | 0.008 | . |
| Government (mean) _(O)_ | 0.047 | . | | **0.206** | **.** | | 0.271 | . |
| COVID-19 symptoms _(N)_ | . | . | | **.** | **.** | | . | . |
| COVID-19 infection _(N)_ | . | . | | **.** | **.** | | . | . |
| *Intercept, R^2^* | *-0.537, 0.169* | *-0.405, 0.126* | | ***20.41, 0.175*** | ***21.01, 0.129*** | | *21.994, 0.167* | *22.821, 0.127* |
| **Regression 2** |  |  | |  |  | |  |  |
| AC Stress F1_(F)_ | -0.311 | -0.242 | | -1.283 | -0.982 | | -1.598 | -1.251 |
| AC Satisfaction F2 _(F)_ | 0.107 | 0.028 | | 0.387 | 0.049 | | 0.524 | 0.137 |
| Worry mean (individual) _(O)_ | -0.036 | . | | -0.145 | . | | -0.175 | . |
| Worry mean (network) _(O)_ | . | . | | . | . | | . | . |
| Worry scale (hospital) _(O)_ | . | . | | . | . | | . | . |
| Adherence scale _(O)_ | . | . | | 0.002 | . | | 0.012 | . |
| Government (mean) _(O)_ | 0.039 | . | | 0.167 | . | | 0.224 | . |
| COVID-19 symptoms _N)_ | . | . | | . | . | | . | . |
| COVID-19 infections _(N)_ | . | . | | . | . | | . | . |
| *Intercept, R^2^* | *-0.357, 0.173* | *-0.404, 0.130* | | *21.162, 0.181* | *21.011, 0.133* | | *22.861, 0.172* | *22.821, 0.132* |

RP1_Reg_a = regression with worry factor scores (5 items, scaling 1-10),

RP1_Reg_b = regression with worry mean and scale scores (scaling 0-10).

AC Stress F1 and AC Satisfaction F2 = Academic stress and satisfaction: high scores for academic stress and satisfaction

F = Factor score, O = ordinal scale, N = nominal scale

**Reported in the manuscript.**

# 2.1. Regression – single variables in SPSS

## Supplementary Table 2. Linear Regression Predicting Mental Well-being with Academic Stress, Satisfaction, COVID-19 related worry, Satisfaction with the government, Adherence, and COVID-19 symptoms and infections

|  | SWEMWBS Factor1 _(COM.)_ | | |  | | SWEMWBS Metric | | |  | | SWEMWBS Raw | | | | |
| --- | --- | --- | --- | --- | --- | --- | --- | --- | --- | --- | --- | --- | --- | --- | --- |
| Predictors | *B (S.E.)* | *Beta (p)* | *R^2^* | |  | *B (S. E.)* | *Beta (p)* | *R^2^* | |  | | *B (S. E.)* | *Beta (p)* | *R^2^* |  |
| **Regression 1** |  |  |  | |  |  |  |  | |  | |  |  |  |  |
| AC Stress F1 _(F)_ | -.397 (.030) | -.380*** | .145 | |  | -1.603 (.120) | -.390*** | .152 | |  | | -2.013 (.154) | -.381*** | .145 |  |
| AC Satisfaction F2 _(F)_ | .318 (.034) | .282*** | .080 | |  | 1.251 (.135) | .282*** | .080 | |  | | -1.597 (.173) | -.280*** | .079 |  |
| Worry F1 _(F)_ | -.127 (.032) | -.124*** | .015 | |  | -.498 (.126) | -.124*** | .015 | |  | | -.581 (.163) | -.112*** | .013 |  |
| Government mean _(O)_ | .132 (.031) | .133*** | .018 | |  | .536 (.122) | .137*** | .019 | |  | | .683 (.157) | .136*** | .019 |  |
| Adherence scale _(O)_ | .029 (.017) | .053 (.096) | .003 | |  | .115 (.068) | .054 (.089) | .003 | |  | | .154 (.087) | .056 (.077) | .003 |  |
| COVID-19 symptoms _(N)_ | -.007 (.047) | -.004 (.887) | .000 | |  | -.031 (.184) | -.005 (.865) | .000 | |  | | -.013 (.237) | -.002 (.957) | .000 |  |
| COVID-19 infection _(N)_ | .028 (.076) | .012 (.715) | .000 | |  | .174 (.301) | .018 (.564) | .000 | |  | | .327 (.387) | .027 (.399) | .001 |  |
| **Regression 2** |  |  |  | |  |  |  |  | |  | |  |  |  |  |
| AC Stress F1 _(F)_ | -.397 (.030) | -.380*** | .145 | |  | -1.603 (.120) | -.390*** | .152 | |  | | -2.013 (.154) | -.381*** | .145 |  |
| AC Satisfaction F2 _(F)_ | .318 (.034) | .282*** | .080 | |  | 1.251 (.135) | .282*** | .080 | |  | | 1.597 (.173) | .280*** | .079 |  |
| Worry mean 1 (individual) _(O)_ | -.048 (.011) | -.138*** | .019 | |  | -.195 (.043) | -.142*** | .020 | |  | | -.234 (.055) | -.132*** | .017 |  |
| Worry mean 2 (network) _(O)_ | -.033 (.012) | -.091** | .008 | |  | -.130 (.046) | -.090** | .008 | |  | | -.146 (.059) | -.079* | .006 |  |
| Worry scale (hospital) _(O)_ | -.030 (.010) | -.093** | .009 | |  | -.111 (.040) | -.088** | .008 | |  | | -.139 (.051) | -.086** | .007 |  |
| Government mean _(O)_ | .132 (.031) | .133*** | .018 | |  | .536 (.122) | -137*** | .019 | |  | | .683 (.157) | .136*** | .019 |  |
| Adherence scale _(O)_ | .029 (.017) | .053 (.096) | .003 | |  | .115 (.068) | .054 (.089) | .003 | |  | | .154 (.087) | .056 (.077) | .003 |  |
| COVID-19 symptoms _(N)_ | -.007 (.047) | -.004 (.887) | .000 | |  | -.031 (.184) | -.005 (.865) | .000 | |  | | -.013 (.237) | -.002 (.957) | .000 |  |
| COVID-19 infection _(N)_ | .028 (.076) | .012 (.715) | .000 | |  | .174 (.301) | .018 (.564) | .000 | |  | | .327 (.387) | .027 (.399) | .001 |  |
| *** p < .001, ** p<.01, *p<.05  RP1_Reg_a = regression with worry factor scores (5 items, scaling 1–10),  RP1_Reg_b = regression with worry mean and scale scores (scaling 0–10).  AC Stress F1 and AC Satisfaction F2 = high scores for academic stress and satisfaction  F = Factor score, O = ordinal scale, N = nominal scale | | | | | | | | | | | | | | | |

## Supplementary Table 3. Means and Standard Deviations for SWEMWBS factor scores by demographic group before and during the COVID-19 outbreak *(See Table 2)*

|  | Before COVID-19 outbreak | |  | During COVID-19 outbreak | |  | ANOVA | | |  | Effect size | | | |
| --- | --- | --- | --- | --- | --- | --- | --- | --- | --- | --- | --- | --- | --- | --- |
| Group | M (SD) | 95 % C.I. |  | M (SD) | 95 % C.I. |  | Adj. mean (S.E.) | 95 % C.I. | p-value |  | | d | CL | |
| Total | 0.03 (0.91) | [0.02, 0.05] |  | -0.40 (0.93) | [-0.46, -0.35] |  | *-* | *-* | *-* | 0.48 | | | | 0.63 |
| Gender |  |  |  |  |  |  | Levene, p < .001 |  | < .001^a^ |  | | | | |
| Male | 0.08 (0.98) | [0.05, 0.12] |  | -0.27 (1.02) | [-0.41, -0.13] |  | -.131 (.021) | [-.172, -.091] |  | 0.36 | | | | 0.60 |
| Female | 0.03 (0.88) | [0.01, 0.05] |  | -0.43 (0.91) | [-0.49, -0.36] |  | -.191 (.016) | [-.222, -.160] |  | 0.52 | | | | 0.64 |
| Other | -0.37 (0.93) | [-0.49, -0.25] |  | -0.99 (0.85) | [-1.46, -0.51] |  | -.602 (.059) | [-.717, -.487] |  | 0.66 | | | | 0.69 |
| Age group |  |  |  |  |  |  | Levene, p = .140 |  | < .001^b^ |  | | | |  |
| 18 – 25 | 0.05 (0.89) | [0.03, 0.07] |  | -0.43 (0.92) | [-0.50, -0.36] |  | -.174 (.017) | [-.207, -.141] |  | 0.54 | | | | 0.65 |
| 26 – 35 | -0.05 (0.92) | [-0.08, -0.02] |  | -0.46 (0.90) | [-0.56, -0.36] |  | -.273 (.019) | [-.311, -.236] |  | 0.44 | | | | 0.62 |
| 36 – 45 | 0.25 (0.93) | [0.20, 0.31] |  | -0.08 (1.04) | [-0.29, 0.13] |  | .040 (.029) | [-.018, .097] |  | 0.35 | | | | 0.59 |
| Education program |  |  |  |  |  |  | Levene, p = .544 |  | .378 |  | | | |  |
| Bachelor | 0.04 (0.91) | [0.02, 0.06] |  | -0.38 (0.92) | [-0.45, -0.31] |  | -.180 (.016) | [-.211, -.148] |  | -0.46 | | | | 0.63 |
| Master | 0.03 (0.92) | [-0.01, 0.06] |  | -0.47 (0.95) | [-0.57, -0.36] |  | -.194 (.020) | [-.234, -.154] |  | -0.54 | | | | 0.65 |
| Doctoral | 0.08 (1.00) | [-0.07, 0.24] |  | 0.17 (1.02) | [-0.52, 0.85] |  | -.102 (.071) | [-.241, .037] |  | 0.07 | | | | 0.48 |
| SWEMWBS factor scores. Effect sizes based on the unstandardized factor scores M (SD).  a. – b. Adjustment for multiple comparisons (Bonferroni):  a. male > female (p = .005), male > other (p < .001), female > other (p < .001);  b. 18-25 > 26-35 (p < .001), 18-25 < 36-45 (p < .001), 26-35 < 36-45 (p < .001)  Total (n):  Before COVID-19 (n = 10 866), During COVID-19 (n = 1 001);  Gender: Male (before: n = 2 948, during: n = 203), Female (before: n = 7 647, during: n = 783), Other (before: n = 240, during: n = 15);  Age groups: 18 – 25 (before: n = 5 908, during: n = 606), 26 – 35 (before: n = 3 884, during: n = 297), 36 – 45 (before: n = 1 074, during: n = 98);  Education program: Bachelor (before: n = 7 762, during: n = 690), Master (before: 2 917, during: n =295), PhD (before: n = 161, during: n = 11)  M = mean, SD = standard deviation, 95 % C.I. = 95 % confidence intervals, Adj. mean (S.E.) = Adjusted mean (standard error), p-values based on the main effects, d = Cohen’s d, CL = Common Language effect size (X>Y) | | | | | | | | | | | | | | |

## Supplementary Table 4. Means and Standard Deviations for SWEMWBS raw scores by Socioeconomic Demographics before and during the COVID-19 outbreak

| Group | Before COVID-19 outbreak | |  | | During COVID-19 outbreak | | | ANOVA | | | | | | | |  | | Effect size | | | | | | | |
| --- | --- | --- | --- | --- | --- | --- | --- | --- | --- | --- | --- | --- | --- | --- | --- | --- | --- | --- | --- | --- | --- | --- | --- | --- | --- |
|  | M (SD) | 95 % C.I. |  |  | M (SD) | | 95 % C.I. |  | | Adj. mean (S.E.) | 95 % C.I. | p-value | | | |  | d | | | | CL | | |  |  |
| Total | 24.95 (4.33) | [24.87, 25.03] | |  | | 22.82 (4.74) | [22.53, 23.11] | | *-* | | *-* | *-* | | | 0.49 | | | | | 0.63 | | | | |  |
| Gender |  |  | |  | |  |  | | Levene, p < .001 | | | | < .001^a^ | | | | | | | | | | | | |
| Male | 25.10 (4.62) | [24.94, 25.27] | |  | | 23.31 (5.00) | [22.62, 24.00] | | 24.06 (.10) | | [23.86, 24.25] | | | 0.39 | | | | | 0.60 | | | |  |  |  |
| Female | 24.96 (4.19) | [24.86, 25.05] | |  | | 22.75 (4.65) | [22.43, 23.08] | | 23.89 (.08) | | [23.74, 24.03] | | | 0.52 | | | | | 0.64 | | |  |  |  |  |
| Other | 22.93 (4.55) | [22.35, 23.51] | |  | | 19.67 (4.59) | [17.12, 22.21] | | 21.80 (.28) | | [21.25, 22.34] | | | 0.72 | | | | | 0.69 | | |  |  |  |  |
| Age group |  |  | |  | |  |  | | Levene, p < .001 | | | | < .001^b^ | | | | | | | | | | | | |
| 18 – 25 | 25.06 (4.23) | [24.95, 25.17] | |  | | 22.73 (4.73) | [22.36, 23.11] | | 24.00 (.08) | | [23.81, 24.12] | | | 0.54 | | | | | 0.64 | | | | |  |  |
| 26 – 35 | 24.51 (4.44) | [24.37, 24.65] | |  | | 22.51 (4.57) | [21.99, 23.03] | | 23.44 (.09) | | [23.26, 23.62] | | | 0.45 | | | | | 0.62 | | | | |  |  |
| 36 – 45 | 25.91 (4.30) | [25.65, 26.17] | |  | | 24.30 (5.07) | [23.28, 25.31] | | 24.87 (.14) | | [24.60, 25.15] | | | 0.37 | | | | | 0.60 | | | | |  |  |
| Education program |  |  | |  | |  |  | | Levene, p = .001 | | | | .529 | | | | | | | | | | | | |
| Bachelor | 24.96 (4.32) | [24.86, 25.05] | |  | | 22.93 (4.68) | [22.58, 23.28] | | 23.91 (.08) | | [23.76, 24.06] | | | -0.47 | | | | | 0.62 | | | | |  |  |
| Master | 24.92 (4.35) | [24.76, 25.08] | |  | | 22.55 (4.80) | [22.00, 23.10] | | 23.85 (.10) | | [23.66, 24.04] | | | -0.54 | | | | | 0.64 | | | | |  |  |
| Doctoral | 25.07 (4.78) | [24.33, 25.82] | |  | | 25.55 (4.50) | [22.52, 28.57] | | 24.19 (.34) | | [23.53, 24.85] | | | 0.10 | | | | | 0.47 | | | | |  |  |
| SWEMWBS raw scores. Effect sizes based on the unstandardized factor scores M (SD).  a. – b. Adjustment for multiple comparisons Bonferroni:  a. male > female (p = .165), male > other (p < .001), female > other (p < .001);  b. 18-25 > 26-35 (p < .001), 18-25 < 36-45 (p < .001), 26-35 < 36-45 (p < .001)  Total (n):  Before COVID-19 (n = 10 866), During COVID-19 (n = 1 001);  Gender: Male (before: n = 2 948, during: n = 203), Female (before: n = 7 647, during: n = 783), Other (before: n = 240, during: n = 15);  Age group: 18 – 25 (before: n = 5 908, during: n = 606), 26 – 35 (before: n = 3 884, during: n = 297), 36 – 45 (before: n = 1 074, during: n = 98);  Education program: Bachelor (before: n = 7 762, during: n = 690), Master (before: 2 917, during: n =295), PhD (before: n = 161, during: n = 11)  M = mean, SD = standard deviation, 95 % C.I. = 95 % confidential intervals, Adj. mean (S.E.) = Adjusted mean (standard error), p-values based on the main effects, d = Cohen’s d, CL = Common Language effect size (X>Y) | | | | | | | | | | | | | | | | | | | | | | | | | |

## Supplementary Table 5. Adjusted Means and Standard Errors for Mental Well-being Standardized by Socioeconomic Demographics before and during the COVID-19 Outbreak

|  | Before COVID-19 | |  | During COVID-19 | | |
| --- | --- | --- | --- | --- | --- | --- |
| *SWEMWBS* | *M (SD)* | *95 % C.I.* |  | *M (SD)* | *95 % C.I.* |  |
| Total_metric_ | 22.7 (3.7) | [22.6, 22.8] |  | 21.0 (3.7) | 20.8 – 21.2 |  |
| Total_factor_ | 0.03 (0.91) | [0.02, 0.05] |  | -0.40 (0.93) | -0.46 – -0.35 |  |
| Total_raw_ | 24.95 (4.33) | [24.87, 25.03] |  | 22.82 (4.74) | 22.53 – 23.11 |  |
|  |  |  |  |  |  |  |
| *Standardized by* | *Adj. mean (S.E.)* | *95 % C.I.* |  | *Adj. mean (S.E.)* | *95 % C.I.* | *p-value* |
| Gender_metric_ | 22.2 (.08) | 22.1 – 22.4 |  | 20.5 (.14) | 20.3 – 20.8 | p < .001 |
| Age_metric_ | 22.9 (.04) | 22.8 – 23.0 |  | 21.2 (.12) | 21.0 – 21.4 | p < .001 |
| Education program _metric_ | 22.8 (.10) | 22.6 – 23.0 |  | 21.1 (.15) | 20.8 – 21.4 | p < .001 |
|  |  |  |  |  |  |  |
| Gender_factor_ | -.09 (.02) | -1.13 – -.05 |  | -.53 (.03) | -.60 – -.46 | p < .001 |
| Age_factor_ | .09 (.01) | .07 – .11 |  | -.36 (.03) | -.42 – -.30 | p < .001 |
| Education program_factor_ | .06 (.02) | .01 – .11 |  | -.38 (.04) | -.45 – -.30 | p < .001 |
|  |  |  |  |  |  |  |
| Gender_raw_ | 24.3 (.10) | 24.1 – 24.5 |  | 22.2 (.16), | 21.9 – 22.5 | p < .001 |
| Age_raw_ | 25.2 (.05) | 25.1 – 25.3 |  | 23.0 (.14), | 22.7 – 23.3 | p < .001 |
| Education program _raw_ | 25.0 (.12) | 24.8 – 25.3 |  | 22.9 (.28), | 22.6 – 23.3 | p < .001 |

# Short Warwick-Edinburgh Mental Well-being Scale (SWEMWBS)

## Supplementary Table 6. Mental Well-being (%)

|  |  |  |  |  |  |  |  |
| --- | --- | --- | --- | --- | --- | --- | --- |
| SWEMWBS items | Dataset | None of the time | Rarely | Some of the time | Often | All of the time | Total  % (N) |
| Item 1: I’ve been feeling optimistic about the future | Before | 1.4 | 8.7 | 28.5 | 48.6 | 12.8 | 100 (10 866) |
|  | *During* | *2.9* | *17.7* | *38.6* | *35.9* | *5.0* | *100 (1 001)* |
|  | **Combined** | **1.5** | **9.5** | **29.4** | **47.6** | **12.1** | **100 (11 867)** |
|  |  |  |  |  |  |  |  |
| Item 2: I’ve been feeling useful | Before | 1.4 | 11.1 | 31.2 | 47.2 | 9.2 | 100 (10 866) |
|  | *During* | *3.2* | *24.7* | *40.2* | *27.3* | *4.7* | *100 (1 001)* |
|  | **Combined** | **1.6** | **12.2** | **31.9** | **45.5** | **8.8** | **100 (11 867)** |
|  |  |  |  |  |  |  |  |
| Item 3: I’ve been feeling relaxed | Before | 3.1 | 26.5 | 40.2 | 27.5 | 2.7 | 100 (10 866) |
|  | *During* | *4.4* | *26.8* | *41.3* | *25.7* | *1.9* | *100 (1 001)* |
|  | **Combined** | **3.2** | **26.5** | **40.3** | **27.4** | **2.7** | **100 (11 867)** |
|  |  |  |  |  |  |  |  |
| Item 4: I’ve been dealing with problems well | Before | 1.5 | 14.0 | 36.0 | 42.1 | 6.3 | 100 (10 866) |
|  | *During* | *3.5* | *19.3* | *38.8* | *33.9* | *4.6* | *100 (1 001)* |
|  | **Combined** | **1.7** | **14.5** | **36.2** | **41.4** | **6.2** | **100 (11 867)** |
|  |  |  |  |  |  |  |  |
| Item 5: I’ve been thinking clearly | Before | 0.7 | 7.3 | 28.0 | 54.1 | 9.9 | 100 (10 866) |
|  | *During* | *1.6* | *12.3* | *32.5* | *45.3* | *8.4* | *100 (1 001)* |
|  | **Combined** | **0.8** | **7.7** | **28.4** | **53.3** | **9.8** | **100 (11 867)** |
|  |  |  |  |  |  |  |  |
| Item 6: I’ve been feeling close to other people | Before | 1.9 | 10.8 | 22.5 | 45.1 | 19.7 | 100 (10 866) |
|  | *During* | *5.8* | *19.7* | *29.2* | *35.5* | *9.9* | *100 (1 001)* |
|  | **Combined** | **2.2** | **11.6** | **23.0** | **44.3** | **18.9** | **100 (11 867)** |
|  |  |  |  |  |  |  |  |
| Item 7: I’ve been able to make up my own mind about things | Before | 0.3 | 3.4 | 15.6 | 50.1 | 30.5 | 100 (10 866) |
|  | *During* | *1.3* | *10.0* | *22.9* | *46.4* | *19.5* | *100 (1 001)* |
|  | **Combined** | **0.4** | **4.0** | **16.2** | **49.8** | **29.6** | **100 (11 867)** |
| SWEMWBS raw scores. Before COVID-19 outbreak (N = 10 866), *During COVID-19 outbreak (N = 1 001)*, **Combined dataset, N = 11 867** | | | | | | | |

## Supplementary Table 7. Correlations Matrix for Mental Well-being before COVID-19 Outbreak, Spearman’s rho

| Variable | Item 1 | Item 2 | Item 3 | Item 4 | Item 5 | Item 6 |
| --- | --- | --- | --- | --- | --- | --- |
| Item 1: I’ve been feeling optimistic about the future | . |  |  |  |  |  |
| Item 2: I’ve been feeling useful | .667** | . |  |  |  |  |
| Item 3: I’ve been feeling relaxed | .401** | .381** | . |  |  |  |
| Item 4: I’ve been dealing with problems well | .484** | .487** | .467** | . |  |  |
| Item 5: I’ve been thinking clearly | .454** | .451** | .444** | .563** | . |  |
| Item 6: I’ve been feeling close to other people | .415** | .436** | .306** | .353** | .346** | . |
| Item 7: I’ve been able to make up my own mind about things | .423** | .434** | .272** | .432** | .476** | .365** |

SWEMWBS **raw scores**. ** Correlation is significant at the 0.01 level (2-tailed). Before COVID-19 outbreak, N = 10 866

## Supplementary Table 8. Correlations Matrix for Mental Well-being during COVID-19 Outbreak, Spearman’s rho

| Variable | Item 1 | Item 2 | Item 3 | Item 4 | Item 5 | Item 6 |
| --- | --- | --- | --- | --- | --- | --- |
| Item 1: I’ve been feeling optimistic about the future | . |  |  |  |  |  |
| Item 2: I’ve been feeling useful | .601** | . |  |  |  |  |
| Item 3: I’ve been feeling relaxed | .447** | .404** | . |  |  |  |
| Item 4: I’ve been dealing with problems well | .515** | .482** | .509** | . |  |  |
| Item 5: I’ve been thinking clearly | .454** | .463** | .442** | .597** | . |  |
| Item 6: I’ve been feeling close to other people | .399** | .424** | .368** | .388** | .376** | . |
| Item 7: I’ve been able to make up my own mind about things | .423** | .403** | .323** | .466** | .579** | .376** |

SWEMWBS **raw scores**. ** Correlation is significant at the 0.01 level (2-tailed). During COVID-19 outbreak N = 1 001

## Supplementary Table 9. Correlations Matrix for Mental Well-being Combined Dataset, Spearman’s rho

| Variable | Item 1 | Item 2 | Item 3 | Item 4 | Item 5 | Item 6 |
| --- | --- | --- | --- | --- | --- | --- |
| Item 1: I’ve been feeling optimistic about the future | . |  |  |  |  |  |
| Item 2: I’ve been feeling useful | .669** | . |  |  |  |  |
| Item 3: I’ve been feeling relaxed | .403** | .382** | . |  |  |  |
| Item 4: I’ve been dealing with problems well | .490** | .489** | .471** | . |  |  |
| Item 5: I’ve been thinking clearly | .458** | .455** | .444** | .567** | . |  |
| Item 6: I’ve been feeling close to other people | .422** | .446** | .312** | .361** | .353** | . |
| Item 7: I’ve been able to make up my own mind about things | .430** | .438** | .276** | .438** | .488** | .373** |

SWEMWBS **raw scores**. ** Correlation is significant at the 0.01 level (2-tailed). Combined dataset N = 11 867

## Supplementary Table 10. Item Factor Analysis (IFA) of Short Warwick-Edinburgh Mental Well-being Scale (SWEMWBS)

| Measure | One-factor model | Two-factor model |
| --- | --- | --- |
| SWEMWBS – Before COVID-19 outbreak |  |  |
| RMSEA (90 % C.I.) | 0.138 (0.133 – 0.142) | 0.070 (0.064 – 0.075) |
| CFI / TLI | 0.960 / 0.940 | 0.994 / 0.985 |
| N | 10 0866 | 10 866 |
| SWEMWBS – During COVID-19 outbreak |  |  |
| RMSEA (90 % C.I.) | 0.131 (0.118 – 0.146) | 0.084 (0.066 – 0.104) |
| CFI / TLI | 0.963 / 0.994 | 0.991 |
| N | 1 001 | 0.977 |
| SWEMWBS – Combined dataset |  |  |
| RMSEA | **0.139 (0.135 – 0.143)** | 0.073 (0.068 – 0.079) |
| CFI / TLI | **0.960 / 0.940** | 0.994/ 0.983 |
| N | **11 867** | 11 867 |
| \| Before COVID-19 outbreak = Student Barometer 2019, N = 10 866;  During COVID-19 outbreak = C19 ISWS (not weighted), N = 1 001;  Combined dataset, N = 11 867;  **Factor analysis used in the manuscript.** \| \| --- \| | | |

## Supplementary Table 11. One-factor Model Item Factor Analysis (IFA) Factor Loadings for SWEMWBS items

| One-factor Model (SWEMWBS) | Before COVID-19 outbreak | During COVID-19 outbreak | **Combined dataset** |
| --- | --- | --- | --- |
| Item 1: I’ve been feeling optimistic about the future | 0.826 (.004) | 0.761 (.017) | **0.825 (.004)** |
| Item 2: I’ve been feeling useful | 0.833 (.004) | 0.751 (.017) | **0.830 (.004)** |
| Item 3: I’ve been feeling relaxed | 0.617 (.007) | 0.657 (.021) | **0.617 (.006)** |
| Item 4: I’ve been dealing with problems well | 0.772 (.005) | 0.804 (.014) | **0.775 (.005)** |
| Item 5: I’ve been thinking clearly | 0.753 (.005) | 0.804 (.015) | **0.758 (.005)** |
| Item 6: I’ve been feeling close to other people | 0.583 (.006) | 0.605 (.023) | **0.593 (.006)** |
| Item 7: I’ve been able to make up my own mind about things | 0.671 (.006) | 0.710 (.019) | **0.679 (.006)** |
| One-factor model. Model results: Estimate (S.E.)  Before COVID-19 outbreak = Student Barometer 2019, N = 10 866;  During COVID-19 outbreak = C19 ISWS (not weighted), N = 1 001;  Combined datasets: N = 11 867  **Factor analysis used in the manuscript.** | | | |

## Supplementary Table 12. Two-factor Model Item Factor Analysis (IFA) Factor Loadings for SWEMWBS items

|  | Before COVID-19 outbreak | | |  | During COVID-19 outbreak | | |  | Combined dataset | |
| --- | --- | --- | --- | --- | --- | --- | --- | --- | --- | --- |
| SWEMWBS | Factor 1 *Est. (S.E.)* | | Factor 2 *Est. (S.E.)* |  | Factor 1 *Est. (S.E.)* | Factor 2 *Est. (S.E.)* | |  | Factor 1 *Est. (S.E.)* | Factor 2 *Est. (S.E.)* |
| Item 1 | ***0.761 (.030)*** | 0.106 (.030) | | ***0.885 (.050)*** | | -0.067 (.055) | ***0.780 (.026)*** | | | 0.088 (.027) |
| Item 2 | ***0.905 (.007)*** | -0.005 (.001) | | ***0.785 (.030)*** | | 0.015 (.021) | ***0.904 (.006)*** | | | -0.009 (.003) |
| Item 3 | 0.030 (.025) | ***0.615 (.023)*** | | ***0.449 (.058)*** | | 0.259 (.061) | 0.124 (.035) | | | ***0.542 (.029)*** |
| Item 4 | 0.028 (.027) | ***0.779 (.024)*** | | *0.414 (.058)* | | *0.450 (.059)* | 0.145 (.043) | | | ***0.691 (.035)*** |
| Item 5 | -0.163 (.022) | ***0.960 (.009)*** | | -0.003 (.002) | | ***0.932 (.027)*** | -0.031 (.033) | | | ***0.867 (.020)*** |
| Item 6 | ***0.374 (.027)*** | 0.248 (.022) | | ***0.491 (.054)*** | | 0.157 (.057) | ***0.423 (.022)*** | | | 0.218 (.022) |
| Item 7 | 0.177 (.027) | ***0.530 (.024)*** | | 0.181 (.068) | | ***0.586 (.066)*** | 0.248 (.033) | | | ***0.487 (.027)*** |
| Item 1: I’ve been feeling optimistic about the future;  Item 2: I’ve been feeling useful;  Item 3: I’ve been feeling relaxed;  Item 4: I’ve been dealing with problems well;  Item 5: I’ve been thinking clearly;  Item 6: I’ve been feeling close to other people;  Item 7: I’ve been able to make up my own mind about things | | | | | | | | | | |

***Highest Factor loading estimates per item shown in bold***

## Supplementary Table 13. Means and Standard Deviations for SWEMWBS raw, metric and factor scores

| Dataset | SWEMWBS | *M (SD)* | *C.I. 95 %* | *Mdn (IQR)* | *25, 75* | *Min, Max* | *n* |
| --- | --- | --- | --- | --- | --- | --- | --- |
| Before COVID-19 outbreak | Raw score | 24.95 (4.33) | 24.87 – 25.03 | 25.00 (6.00) | 22, 28 | 7, 35 | 10 866 |
|  | Metric score | 22.69 (3.68) | 22.62 – 22.76 | 22.35 (5.05) | 19.98, 25.03 | 7, 35 | 10 866 |
|  | Factor score | 0.03 (0.91) | 0.017 – 0.051 | 0.079 (1.178) | -0.57, 0.61 | -3.52, 2.54 | 10 866 |
| During COVID-19 outbreak | Raw score | 22.82 (4.74) | 22.53 – 23.11 | 23.00 (6.00) | 20, 26 | 7, 35 | 1 001 |
|  | Metric score | 21.01 (3.68) | 20.78 – 21.24 | 20.73 (4.62) | 18.59, 23.21 | 7, 35 | 1 001 |
|  | Factor score | -0.40 (0.93) | -0.46 – -0.35 | -0.429 (1.288) | -1.04, 2.54 | -3.52, 2.54 | 1 001 |
| Combined dataset | Raw score | 24.77 (4.01) | 24.69 – 24.85 | 25.00 (6.00) | 22, 28 | 7, 35 | 11 867 |
|  | Metric score | 22.55 (3.71) | 22.48 – 22.62 | 22.35 (5.05) | 19.98, 22.35 | 7, 35 | 11 867 |
|  | Factor score | -0.003 (0.92) | -0.20 – 0.014 | 0.03 (1.24) | -0.63, 0.03 | -3.52, 2.54 | 11 867 |

Factor scores based on the one-factor model

# 3.2 Measures of Academic Stress and Satisfaction

## Supplementary Table 14. Satisfaction towards higher education institute during COVID-19 outbreak (%)

|  | *Strongly agree* | *Agree* | *Neither agree nor disagree* | *Disagree* | *Strongly disagree* | *Total %* |
| --- | --- | --- | --- | --- | --- | --- |
| 1. My university/college workload has significantly increased since the COVID-19 outbreak. | 16.4 | 23.1 | 27.1 | 24.2 | 9.3 | 100 |
| 1. I know less about what is expected of me in the different course modules/units since the COVID-19 outbreak. | 13.6 | 30.3 | 21.0 | 25.5 | 9.7 | 100 |
| 1. I am concerned that I will not be able to successfully complete the academic year due to the COVID-19 outbreak. | 9.4 | 18.1 | 14.1 | 29.9 | 28.6 | 100 |
| 1. The university/college provides poorer quality of education during the COVID-19 outbreak as before. | 12.2 | 21.3 | 29.9 | 26.9 | 9.8 | 100 |
| 1. The change in teaching methods resulting from the COVID-19 outbreak has caused me significant stress. | 16.2 | 24.8 | 20.1 | 22.3 | 16.7 | 100 |
| 1. The university/college has sufficiently informed me about the changes that were implemented due to the COVID-19 outbreak. | 31.2 | 46.7 | 11.1 | 9.3 | 1.8 | 100 |
| 1. I am satisfied with the way my university/college has implemented protective measures concerning the COVID-19 outbreak. | 35.9 | 42.0 | 15.4 | 5.0 | 1.8 | 100 |
| 1. I feel I can talk to a member of the university/college staff (e.g., professor, student counsellor) about my concerns due to the COVID-19 outbreak. | 12.3 | 29.0 | 33.2 | 17.0 | 8.6 | 100 |
| During COVID-19 outbreak, N = 1 001 (not weighted) | | | | | | |

## Supplementary Table 15. Correlation Matrix for Mental Well-being and Academic Stress and Satisfaction, Spearman’s rho

| Variable | 1 | 2 | 3 | 4 | 5 | 6 | 7 |
| --- | --- | --- | --- | --- | --- | --- | --- |
| Item 1. (Stress) | . |  |  |  |  |  |  |
| Item 2. (Stress) | .393** | . |  |  |  |  |  |
| Item 3. (Stress) | .269** | .381** | . |  |  |  |  |
| Item 4. (Stress) | .272** | .509** | .345** | . |  |  |  |
| Item 5. (Stress) | .444** | .503** | .471** | -.539** | . |  |  |
| Item 6. (Satisfaction) | -.105** | -.218** | -.208** | -.273** | -.211** | . |  |
| Item 7. (Satisfaction) | -.098** | -.196** | -.186** | -.298** | -.221** | .536** | . |
| Item 8. (Satisfaction) | -.100** | -.220** | -.194** | -.185** | -.173** | .262** | .270** |
| SWEMWBS raw scores. Correlation is significant at the 0.01 level (2-tailed)  1. My university/college workload has significantly increased since the COVID-19 outbreak.  2. I know less about what is expected of me in the different course modules/units since the COVID-19 outbreak.  3. I am concerned that I will not be able to successfully complete the academic year due to the COVID-19 outbreak.  4. The university/college provides poorer quality of education during the COVID-19 outbreak as before.  5. The change in teaching methods resulting from the COVID-19 outbreak has caused me significant stress.  6. The university/college has sufficiently informed me about the changes that were implemented due to the COVID-19 outbreak.  7. I am satisfied with the way my university/college has implemented protective measures concerning the COVID-19 outbreak.  8. I feel I can talk to a member of the university/college staff (e.g., professor, student counsellor) about my concerns due to the COVID-19 outbreak.  During COVID-19 outbreak, N = 1 001 | | | | | | | |

## Supplementary Table 16. Item Factor Analysis (IFA) for Academic stress and satisfaction

| Measure | One-factor model | **Two-factor model** |
| --- | --- | --- |
| Academic Stress and Satisfaction |  |  |
| RMSEA (90 % C.I.) | 0.186 (.175 - .198) | **0.066 (.051 - .082)** |
| CFI / TLI | 0.828 / 0.760 | **0.986 / 0.970** |
| N | 1 001 | **1 001** |
| High scores indicate higher Academic Stress and Satisfaction. Model used in the analysis  **Model used in the manuscript.** | | |

## Supplementary Table 17. Item Factor Analysis (IFA) Factor Loadings for Academic stress and satisfaction

|  | 1-factor IFA  *Est. (S.E.)* | 2-factor IFA | |
| --- | --- | --- | --- |
|  |  | Factor 1  *Est. (S.E.)* | Factor 2  *Est. (S.E.)* |
| Item 1 (Stress) | .502 (.025) | ***.618 (.031)*** | .133 (.039) |
| Item 2 (Stress) | .711 (.020) | ***.739 (.021)*** | -.009 (.014) |
| Item 3 (Stress) | .595 (.024) | ***.575 (.032)*** | -.073 (.041) |
| Item 4 (Stress) | .727 (.018) | ***.639 (.026)*** | -.180 (.032) |
| Item 5 (Stress) | .778 (.017) | ***.832 (.022)*** | .017 (.029) |
| Item 6 (Satisfaction) | -.560 (.027) | -.018 (.043) | ***.741 (.047)*** |
| Item 7 (Satisfaction) | -.572 (.025) | .010 (.008) | ***.829 (.038)*** |
| Item 8 (Satisfaction) | -.370 (.030) | -.131 (.042) | ***.356 (.041)*** |

***Highest factor loadings for each factor shown in bold***

1. My university/college workload has significantly increased since the COVID-19 outbreak

2. I know less about what is expected of me in the different course modules/units since the COVID-19 outbreak.

3. I am concerned that I will not be able to successfully complete the academic year due to the COVID-19 outbreak.

4. The university/college provides poorer quality of education during the COVID-19 outbreak as before.

5. The change in teaching methods resulting from the COVID-19 outbreak has caused me significant stress

6. The university/college has sufficiently informed me about the changes that were implemented due to the COVID-19 outbreak.

7. I am satisfied with the way my university/college has implemented protective measures concerning the COVID-19 outbreak.

8. I feel I can talk to a member of the university/college staff (e.g., professor, student counsellor) about my concerns due to the COVID-19 outbreak.

# 3.3 Measures/Scales: COVID-19 related worry

## Supplementary Table 18. Distributions for COVID-19 related worry items (%)

|  | *M (SD)* | *95 % C.I.* | *Mdn (IQR)* | *25%, 75%* |
| --- | --- | --- | --- | --- |
| Item 1. How worried are you to get (re-)infected with COVID-19? | 4.27 (2.78) | 4.09 – 4.44 | 4.00 (4.00) | 2, 6 |
| Item 2. How worried are you that you will get severely ill from a  COVID-19 (re-)infection? | 4.42 (2.99) | 4.23 – 4.61 | 4.00 (5.00) | 2, 7 |
| Item 3. How worried are you that anyone from your personal  network will get infected with COVID-19? | 6.68 (2.63) | 6.51 – 6.84 | 7.00 (4.00) | 5, 9 |
| Item 4. How worried are you that anyone from your personal  network will get severely ill from a COVID-19 infection? | 7.14 (2.69) | 6.97 – 7.31 | 8.00 (3.00) | 6, 9 |
| Item 5. How worried are you that doctors and hospitals will not have  sufficient medical supplies to handle the COVID-19  outbreak? | 5.29 (2.92) | 5.11 – 5.47 | 5.00 (5.0) | 3, 8 |
| Scaling 0-10, 0 = not worried, 10 = very worried.  During COVID-19 outbreak N = 1 001 (not weighted)  M = mean, SD = Standard deviation, 95 % C.I = 95 % Confidence interval, Mdn = median, IQR = interquartile range, 25 = lower percentiles, 75 = upper percentiles, Scaling 0-10, 0 = not worried at all, 10 = very worried. | | | | |

## Supplementary Table 19. Correlations Matrix for COVID-19 Related Worry, Spearman’s rho

| Variable | 1 | 2 | 3 | 4 |
| --- | --- | --- | --- | --- |
| Item 1. | . |  |  |  |
| Item 2. | .719** | . |  |  |
| Item 3. | .590** | .453** | . |  |
| Item 4. | .533** | .522** | .826** | . |
| Item 5. | .451** | .397** | .474** | .446** |
| Correlation is significant at the 0.01 level (2-tailed)  Scaling 0-10, 0 = not worried, 10 = very worried. During COVID-19 outbreak, N = 1 001 | | | | |

Item 1. How worried are you to get (re-)infected with COVID-19?

Item 2. How worried are you that you will get severely ill from a COVID-19 (re-)infection?

Item 3. How worried are you that anyone from your personal network will get infected with COVID-19?

Item 4. How worried are you that anyone from your personal network will get severely ill from a COVID-19 infection?

Item 5. How worried are you that doctors and hospitals will not have sufficient medical supplies to handle the COVID-19 outbreak?

## Supplementary Table 20. Item Factor Analysis (IFA) fit for COVID-19-related worry

| Measure | **One-factor model** |
| --- | --- |
| COVID-19 related worry |  |
| RMSEA (90 % C.I.) | **0.329 (.306 - .353)** |
| CFI / TLI | **0.958 / 0.915** |
| N | **1 001** |

## Supplementary Table 21. Item Factor Analysis (IFA) standardized factor loadings for COVID-19-related worry

|  | Est. (S.E.) |
| --- | --- |
| Item 1: How worried are you to get (re-)infected with COVID-19? | 0.810 (.011) |
| Item 2: How worried are you that you will get severely ill from a COVID-19 (re-)infection? | 0.772 (.013) |
| Item 3: How worried are you that anyone from your personal network will get infected with COVID-19? | 0.908 (.008) |
| Item 4: How worried are you that anyone from your personal network will get severely ill from a COVID-19 infection | 0.910 (.008) |
| Item 5: How worried are you ... - ... that doctors and hospitals will not have sufficient medical supplies to handle the COVID-19 outbreak? | 0.558 (.021) |

# 3.4 Measures of Government satisfaction

## Supplementary Table 22. Satisfaction with government during COVID-19 outbreak (%)

|  | Strongly agree | Agree | Neither agree nor disagree | Disagree | Strongly disagree | Total % |
| --- | --- | --- | --- | --- | --- | --- |
| The government provided information concerning the COVID-19 outbreak on time | 24.7 | 46.4 | 13.8 | 12.8 | 2.4 | 100 |
| The government provided comprehensive information regarding COVID-19 outbreak | 26.9 | 48.0 | 12.1 | 10.9 | 2.2 | 100 |
| During COVID-19 outbreak, N = 1 001. Correlation between the two items is 742, p<.001**  Single items (0=Strongly disagree, 4=Strongly agree):  (a) on time: Mean=2.78, SD=1.03, 95% C.I=2.72-2.85, Mdn=3.0, IQR=1;  (b) comprehensive: Mean=2.86, SD=1.00, 95% C.I.=2.80-2.93, Mdn=2.0, IQR=2  (c) combined (mean): Mean=2.82, SD=0.94, 95% C.I.=2.76-2.88, Mdn=3.0, IQR=1.50 | | | | | | |

# 3.5 Correlations between Mental Well-Being and COVID-19 Related Questions

## Supplementary Table 23. Correlation Matrix for Mental Well-being and COVID-19 Related Questions

| Variable | 1 | 2 | 3 | 4 | 5 | 6 | 7 | 8 | 9 | 10 |
| --- | --- | --- | --- | --- | --- | --- | --- | --- | --- | --- |
| 1. SWEMWBS (F) | . |  |  |  |  |  |  |  |  |  |
| 2. SWEMWBS (M) | .989** | . |  |  |  |  |  |  |  |  |
| 3. SWEMWBS (R) | .989** | 1.00** | . |  |  |  |  |  |  |  |
| 4. Worry 1 (mean) | -.126** | -.125** | -.125** | . |  |  |  |  |  |  |
| 5. Worry 2 (mean) | -.084** | -.081** | .081** | .587** | . |  |  |  |  |  |
| 6. Worry 3 (R) | -.086** | -.079* | -.079** | .453** | .477** | . |  |  |  |  |
| 7. Worry (F) | -.105** | -.103** | -.103** | .781** | .958** | .563** | . |  |  |  |
| 8. Gov. (mean) | .133** | .136** | .136** | -.120** | -.055 | -.133** | -.091** | . |  |  |
| 9. AC Stress F1 (F) | -.363** | -.371** | -.371** | .020 | .039 | .098** | .042 | -.097** | . |  |
| 10. AC Satisfaction F2 (F) | .277** | .274** | .274** | .011 | .014 | -.047 | .010 | .204** | -.496** |  |
| 11. Adherence (R) | .052 | .048 | .048 | .202** | .209** | .144** | .229** | .106** | -.128** | .116** |
| ** Correlation is significant at the 0.01 level (2-tailed)  * Correlation is significant at the 0.05 level (2-tailed)  F = Factor score, M = Metric score, R = Raw score  Worry 1 = individual worry: 2 items  Worry 2 = network worry: 2 items  Worry 3 = hospital worry  Worry = Factor score based on 5 items  Gov. = Government satisfaction  AC = Academic Stress and Satisfaction | | | | | | | | | |  |

# 4. Similarities between the two cross-sectional samples based on top 10 higher education institutes (before, during COVID-19)

## Supplementary Table 24. Participation numbers by institute in the two samples and total enrolled students

| Before COVID-19 outbreak  (n=10 866) | | | During COVID-19 outbreak (n=1 001) | | | Student totals  Vipunen 2019 | | |
| --- | --- | --- | --- | --- | --- | --- | --- | --- |
| *TOP 10 HEI* | *n* | *%* | *TOP 10 HEI* | *n* | *%* | *TOP 10 HEI* | *n* | *%* |
| University of Eastern Finland | 1063 | 9.8 | University of Eastern Finland | 233 | 23.3 | University of Helsinki | 31 476 | 10.6 |
| University of Turku | 977 | 9.0 | Karelia UAS | 161 | 16.1 | University of Tampere | 20 868 | 7.0 |
| University of Helsinki | 838 | 7.7 | University of the Arts Helsinki | 116 | 11.6 | Aalto University | 17 625 | 5.9 |
| University of Jyväskylä | 808 | 7.4 | Aalto University | 111 | 11.1 | Helsinki Metropolia UAS | 16 245 | 5.5 |
| Metropolia UAS | 741 | 6.8 | Diaconia UAS | 59 | 5.9 | University of Turku | 16 200 | 5.4 |
| Tampere University | 634 | 5.8 | Oulu UAS | 43 | 4.3 | University of Eastern Finland | 15 525 | 5.2 |
| Aalto University | 584 | 5.4 | Humak UAS | 41 | 4.1 | University of Jyväskylä | 13 809 | 4.6 |
| Tampere UAS | 486 | 4.5 | University of Helsinki | 31 | 3.1 | University of Oulu | 12 546 | 4.2 |
| Turku UAS | 469 | 4.3 | Tampere University | 30 | 3.0 | Haaga-Helia UAS | 10 620 | 3.6 |
| University of Oulu | 437 | 4.0 | Turku UAS,  Haaga-Helia UAS | 26  26 | 2.6  2.6 | Turku UAS | 10 311 | 3.5 |
| HEI: Higher education institute, UAS: University of Applied Sciences  List of students at different universities in 2019: <https://vipunen.fi/en-gb/university/Pages/Opiskelijat-ja-tutkinnot.aspx>  List of students at different UAS in 2019: https://vipunen.fi/en-gb/polytechnic/Pages/Opiskelijat-ja-tutkinnot.aspx | | | | | | | | |
